# Serum vitamin D levels are associated with acute postoperative pain and opioid analgesic consumption after laparoscopic cholecystectomy: a strobe compliant prospective observational study

**DOI:** 10.55730/1300-0144.5570

**Published:** 2022-11-20

**Authors:** Ökkeş Hakan MİNİKSAR, Ahmet YÜKSEK, Ayşe Yeşim GÖÇMEN, Mehmet Kağan KATAR, Mahmut KILIÇ, Mehtap HONCA

**Affiliations:** 1 Department of Anesthesiology and Reanimation, Faculty of Medicine, Yozgat Bozok University, Yozgat, Turkey; 2 Department of Biochemistry, Faculty of Medicine, Yozgat Bozok University, Yozgat, Turkey; 3 Department of General Surgery, Faculty of Medicine, Yozgat Bozok University, Yozgat, Turkey; 4 Department of Public Health, Faculty of Medicine, Yozgat Bozok University, Yozgat, Turkey

**Keywords:** Vitamin D, vitamin D deficiency, postoperative pain, analgesia, laparoscopy

## Abstract

**Background/aim:**

In this prospective observational study, we aimed to evaluate the relationship between serum levels of vitamin D and acute postoperative pain scores, as well as opioid analgesic consumption in patients undergoing laparoscopic cholecystectomy.

**Materials and methods:**

The study was performed in the Medical Faculty Hospital, from April 2020 to April 2021. Postoperative visual analog scale (VAS) pain scores, total tramadol consumption, number of requests on patient-controlled analgesia (PCA) were compared between the vitamin D deficient (≤20 ng/mL; n = 25) and vitamin D nondeficient (>20 ng/mL; n = 55) groups at five time points (T0: in the recovery room, T1: 1st hour in the ward, T2: 6th hour, T3: 12th hour, and T4: 24th hour).

**Results:**

Postoperative VAS pain scores were similar in the vitamin D deficient group at all time points (T0–4), but differed significantly only at the T-0 time point (p = 0.020). The mean cumulative tramadol consumption was significantly higher in the vitamin D deficiency group than in the nondeficiency group (p = 0.005). Vitamin D levels were lower in patients with VAS ≥ 4 at the postoperative T-0 time point (p = 0.009). In the multivariate linear regression analysis, 15.7% of cumulative tramadol consumption was due to vitamin D deficiency (β = −0.188).

**Conclusion:**

Our study shows that preoperative low vitamin D level was associated with an increase in acute postoperative pain scores and consumption of opioid analgesics in patients undergoing laparoscopic cholecystectomy. Our findings may be useful for postoperative pain management in patients with vitamin D deficiency.

## 1. Introduction

Cholecystectomy is one of the most common intraabdominal surgical procedures, usually performed laparoscopically. Acute postoperative pain can be observed in 30%–70% of patients after laparoscopic surgery [1]. Many studies have reported increased length of hospital stay, sleep disturbance, decreased patient satisfaction, prolonged immobilization time, and increased use of opioid analgesics due to poor control of acute postoperative pain [1–2].

Vitamin D deficiency is very common in both developed and developing countries. Circulating vitamin D is transported to organs via vitamin D binding protein (DBP) and exerts its effects on organs by binding to intracellular vitamin D receptors (VDR) [3]. Bile acids are required for absorption of vitamin D. Therefore, patients with biliary diseases in which biliary secretion is impaired are at risk of vitamin D deficiency [4–6]. In addition, patients with a history of cholecystectomy have been reported to have lower levels of vitamin D than their healthy peers [7,8].

The relationship between vitamin D and pain is one of the contemporary research topics. Vitamin D deficiency has been associated with various pain syndromes including nonspecific musculoskeletal pain, widespread chronic pain [9], headache [10], and acute postoperative pain [11]. Moreover, both observational and interventional studies have stated that vitamin D plays an important role in pain intensity and pain management [9–12]. Although various mechanisms have been suggested, its exact pathophysiology is not clearly understood. The antiinflammatory and neuroimmunomodulatory action of vitamin D has been proposed to be the most probable mechanisms that can lead to pain sensitization [10–12]. The relationship between vitamin D deficiency and many types of pain has been studied, but its relationship with acute postoperative pain has not been adequately investigated.

There is no effective preoperative biomarker that can help predict the severity of postoperative pain and opioid analgesic consumption. In a recent study, low serum vitamin D levels were reported to be predictive of higher postoperative opioid analgesic consumption in patients that underwent surgical procedures [12]. Determining the relationship between preoperative vitamin D levels and postoperative acute pain scores and opioid consumption in patients undergoing cholecystectomy surgery, which is a risk factor for vitamin D deficiency, may help clinicians’ pain management strategy. To our knowledge, there are no studies examining the effect of vitamin D deficiency on acute postoperative pain after cholecystectomy.

We hypothesized that the serum vitamin D levels correlate with postoperative pain scores and opioid analgesic consumption. The main objective of this study was therefore to evaluate the postoperative pain scores and opioid analgesic consumption according to serum vitamin D levels in patients undergoing laparoscopic cholecystectomy. Secondary objectives were to determine the correlation of vitamin D levels with postoperative pain scores and opioid analgesic consumption, and factors affecting postoperative 24h cumulative opioid analgesic consumption.

## 2. Methods

### 2.1. Study design

This prospective observational study was conducted between April 2020 and April 2021 at Yozgat Bozok University Medical Faculty Hospital, a tertiary care center. The study was approved by the Ethics Committee of the Bozok University, Turkey (protocol number: KAEK-189_2020.03.25_02) (Chairperson Prof S. Dinç) on 25 March 2020 and Helsinki Declaration guidelines were followed throughout the study. All participants signed a written informed consent to participate in the study. The study was recorded at ClinicalTrials.gov with the following ID code: NCT04687527. This prospective observational study was reported as per the guidelines in the STrengthening the Reporting of OBservational studies in Epidemiology (STROBE) statement for observational studies.

### 2.2. Study participants

**Figure 1:**
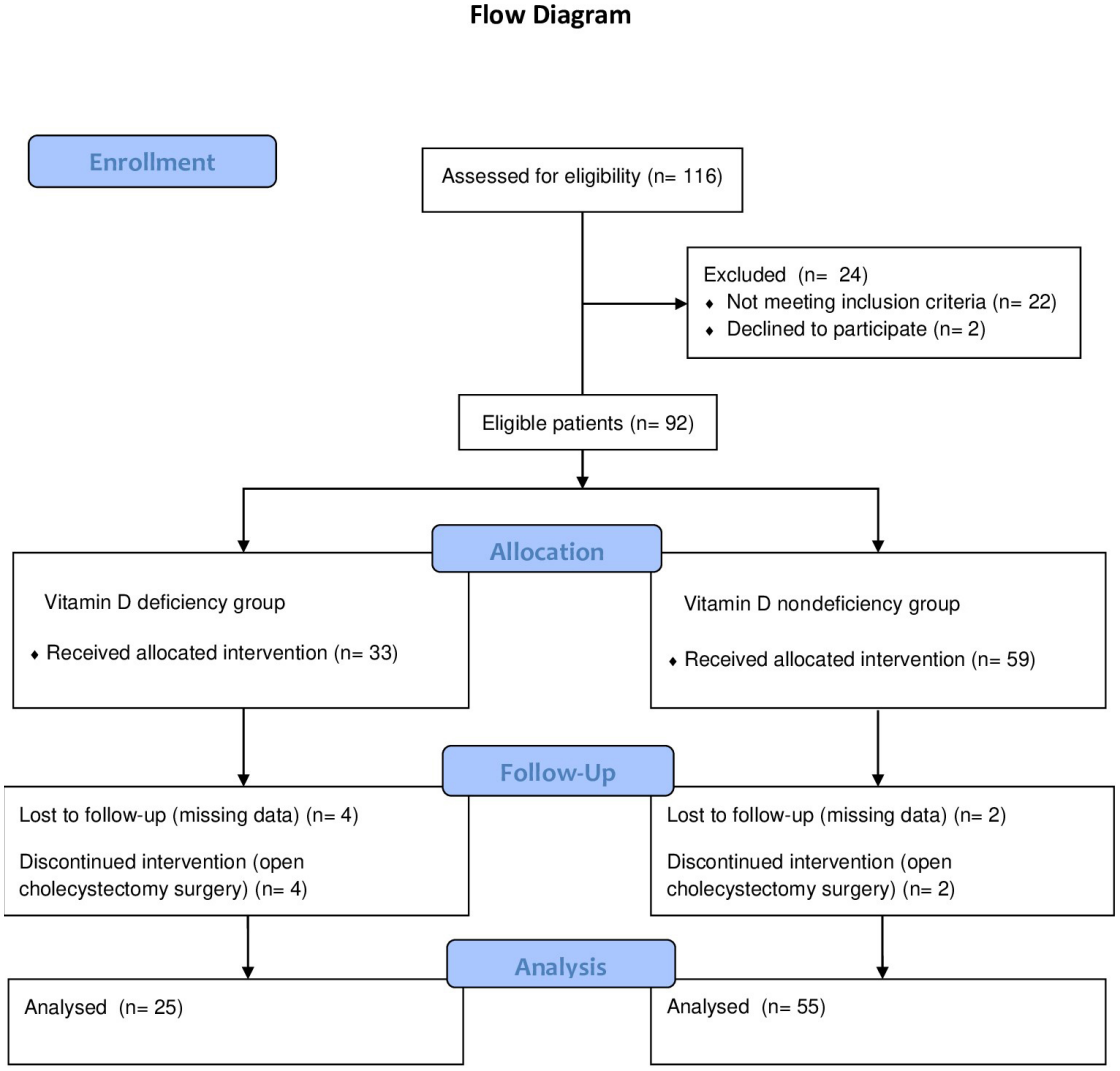
Flow diagram of the study.

A total of 92 adult patients who had undergone laparoscopic cholecystectomy under general anesthesia between the specified dates and met the inclusion criteria were included in the study. The study’s inclusion criteria were: (1) age between 18 and 45 years, (2) being scheduled for elective laparoscopic cholecystectomy, and (3) ASA (American Society of Anesthesiologists) physical status I–III. The exclusion criteria were: (1) history of chronic pain or taking regular analgesics preoperatively, (2) known psychiatric and neurological disease and taking medication to treat those diseases, (3) history of alcohol and substance abuse, (4) hearing and visual deficits, (5) sensitivity to nonsteroidal antiinflammatory drugs (NSAIDs) or opioids, documented history of difficult intubation, (6) moderate to severe obesity (body mass index (BMI) > 35 kg/m2), and (7) emergency surgery (Figure 1). In addition, patients that lacked data on opioid consumption and pain scores, those who could not use a patient-controlled analgesia (PCA) device, those who could not understand the visual analog scale (VAS), and those who used analgesics or sedatives in the 24-hour period before the surgery were excluded from the study. Missing data and discontinued interventions (open cholecystectomy surgery) were excluded from follow-up.

### 2.3. Clinical data

Patients’ demographic data such as age, BMI and ASA physical status were recorded before the operation. Venous blood samples were collected from each patient just before induction of anesthesia and kept frozen at −80°C until analysis. Patients were divided into two groups according to serum 25-hydroxyvitamin D concentration: vitamin D deficiency group (serum level less than ≤ 20 ng/mL) and vitamin D nondeficiency group (serum level > 20 ng/mL) [9,13–15]. In addition, patients were divided into two groups as VAS ≥ 4/10 and VAS < 4/10 at the postoperative T-0 time point. Demographic and clinical characteristics of the patients were compared between the groups.

**Figure 2:**
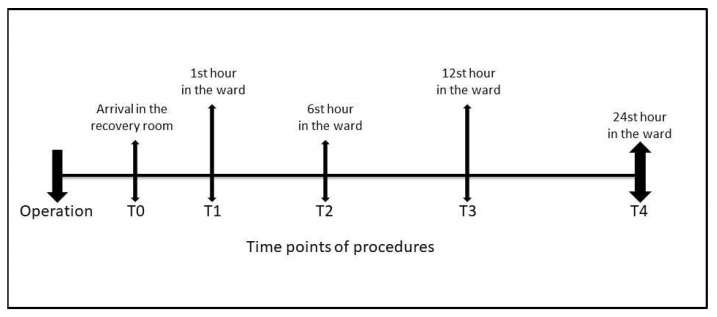
The mean of postoperative pain scores and consumed tramadol doses at different time points according to serum vitamin D level groups.

Data were analyzed at five time points in the postoperative period: T0: in the recovery room, T1: 1st hour in the ward, T2: 6th hour in the ward, T3: 12th hour in the ward, and T4: 24th hour in the ward (Figure 2). Patients’ VAS pain scores, numbers of requests on PCA, and total tramadol consumptions were recorded at all time-points. The primary outcome measure of this study was postoperative pain scores and opioid analgesic consumption according to serum vitamin D levels. Secondary outcome measures included the correlation of vitamin D levels with postoperative pain scores and opioid analgesic consumption, and factors affecting postoperative 24h cumulative opioid analgesic consumption.

### 2.4. Anesthetic and surgery management

None of the patients required sedation and analgesics for premedication purposes. Once the patients were in the operating room, routine monitoring (electrocardiography [EKG], noninvasive blood pressure and pulse oximetry [SpO2]) was performed and an 18-gauge IV cannula was inserted into a vein on the dorsum of the right hand. Crystalloid infusion (8 mL/kg) was started in all patients and continued throughout the surgery. Preoxygenation was performed for 3 min with 100% O2. All patients received standardized anesthesia induction (2–3 mg/kg propofol [Propofol 1%, Fresenius], 0.6–1.2 mg/kg rocuronium [Esmeron, MSD] and 1 μg/kg fentanyl [Talinat, VEM^®^]). After orotracheal intubation, patients were set to maintain a tidal volume of 8 mL/kg, FIO2 0.6, I:E ratio 1:2, respiratory rate 12 breaths/min, ETCO2 30 to 40 mm Hg in a controlled ventilation mode. Maintenance of anesthesia was provided by inhalation of 60% O2 and 40% air and sevoflurane (minimum alveolar concentration [MAC] 0.8%–1.3%). Following intubation, 0.1 mg/kg rocuronium was administered when necessary for muscle relaxation. Additional fentanyl supplementation was used to maintain blood pressure and heart rate within 20% of baseline values intraoperatively. The mean blood pressure was maintained above 60 mm Hg with ephedrine boluses. Bradycardia (heart rate < 45 beats/min) was treated with atropine.

All patients received 10 mg metoclopramide IV, 1.5 mg/kg of tramadol IV (Tramosel 100 mg/mL, HAVER), and 1 g paracetamol IV (Partemol 1g/100mL, VEM) for postoperative nausea and analgesia, 30 min before the end of the operation. At the end of the surgery, residual neuromuscular blockade was antagonized with an IV neostigmine (50 μg/kg) and atropine (20 μg/kg) combination. When the patient was awake, the endotracheal tube was extubated.

All laparoscopic cholecystectomies were managed by the same highly experienced team (surgeons and anesthesiologists). All clinical steps such as anesthetic technique and administration of medications were managed according to standard institutional protocols. As a standard surgical technique, 4 trocars were used: 10 mm from the umbilical region, 5 and 10 mm on the left and 5 mm on the right. The gallbladder was retracted via the subumbilical 10-mm port site. The intraoperative carbondioxide pneumoperitoneum pressure was maintained at 12–14 mm Hg with carbondioxide and this gas was evacuated at the end of surgery.

### 2.5. Postoperative pain management protocol

During the preoperative visit, all patients were informed about the use of the PCA device and VAS for postoperative at rest pain assessment (where 0 = no pain and 10 = worst possible pain). Postoperative pain at the recovery room and the ward was assessed by an investigator using the VAS at 1st, 6th, 12th and 24th h after surgery.

In the recovery room, patients were asked to estimate, on a horizontal VAS ruler (by awake patients), to rate their baseline pain (T-0) between 0 and 10 according to VAS and recorded to one decimal point (0.0–10.0). Pain sensation was evaluated every 10 min, patients with VAS scores of 4–5 were treated with 10 mg IV tramadol bolus, while those with VAS scores > 5 were treated with 20 mg IV tramadol bolus. If VAS scores did not fall below 4 after 50 mg tramadol bolus, rescue analgesia was planned to be provided with 2 mg IV morphine. After 30 min of follow-up in the recovery room, a PCA device (BodyGuard 575 Pain Manager; CME Ltd, New York, NY) containing a tramadol set to a bolus dose of 10 mg, a basal infusion of 10 mg/h, and a 10-minute lock interval was placed in the venous line and the infusion was initiated. IV metoclopramide was given every 8 h for 24 h. Any side effects such as nausea and vomiting were reported and treated. Patients who were hemodynamically stable and had a VAS less than 4 were transferred to the ward after 30 min in the recovery room.

### 2.6. Pain management in the ward

The patients were followed up in a similar way at the 1st, 6th, 12th and 24th hours in the postoperative period. At each time point, VAS pain scores, tramadol requests (number of PCA button presses), total tramadol amount administered to the patient, and cumulative tramadol consumed within 24 h were retrieved from PCA pump memory. The pain management strategy and indication for rescue analgesia were the same as for the recovery room. After the initiation of PCA, the patient’s vitals were closely monitored, and overdose of PCA solution (tramadol) was avoided.

### 2.7. Sample collection and measurement of serum levels of vitamin D, VDR and VDBP

Venous blood was collected from patients just before the induction of anesthesia to measure the serum concentration of vitamin D, VDR and VDBP. Collected blood samples were centrifuged at 3000 rpm for 10 min. Then, the supernatant was removed and kept frozen at −80 °C. Serum vitamin D, VDR and VDBP concentrations were measured with Olympus AU 600 autoanalyzer (Olympus Optical Co., Japan) by using commercial ELISA kits (Elabscience Bioengineering Co., Ltd., Wuhan, China). Serum vitamin D and DBP concentrations were expressed as ng/mL, while VDR concentrations were expressed as pg/mL.

### 2.8. Statistical analysis

The data was analyzed with IBM SPSS Statistics Standard Concurrent User V 25 (IBM Corporation, Armonk, NY, USA). Descriptive statistics were presented as mean (standard deviation), frequency distribution and percentage. The normality of distribution was checked using the Kolmogorov-Smirnov test and histograms. The significance of differences between groups in terms of averages were assessed with chi-square test, independent samples t-test, Mann-Whitney U test, Pearson’s and Spearman’s rho correlation, and linear regression (LR). The effects of categorical variables (dummy variables: sex [male vs. female]) and covariates (age, BMI, surgical time, vitamin D, VAS-T0) as independent variables on postoperative pain score and 24h cumulative tramadol consumption were analyzed using the LR backward model (Adjusted R2) while adjusting for potential confounding factors. Sex (male and female), which are categorical variables, were converted into a dummy variable and included in the regression analysis. The model fit was assessed using appropriate residual and goodness of fit statistics. Variables were the relationships between serum vitamin D levels, VAS pain scores, total tramadol consumption at each time point and 24h cumulative tramadol consumption were examined by a Pearson correlation analysis. All comparative analyzes were 2-tailed, and a *p*-value of less than 0.05 was considered to be statistically significant.

### 2.9. Sample size

The sample size was calculated using the G*Power 3.1 program (version 3.1.9; Kiel, Germany) based on the difference of the arithmetic mean in two independent groups. Hao-Wei Xu et al. [13] found that the mean postoperative VAS pain score (SD) was 4.52 (1.66) in the vitamin D deficit group and 3.50 (1.39) in the nondeficit group. When the difference was at least 1 score, type 1 error of 0.05 (two-sided) and the power (1-β) was 0.90, was calculated the minimum sample size as 46 patients, with at least 23 patients in each group.

## 3. Results

### 3.1. Analysis of clinical variables in terms of study population characteristics and serum vitamin D levels

A total of 80 patients between the ages of 28 to 45 years with a mean (SD) age of 37.9 (4.01) years that underwent elective laparoscopic cholecystectomy and had ASA physical status I–III were included in the study. Among the patients 54 (67.5%) were female. The mean serum vitamin D, DBP and VDR concentrations (SD) were 23.91 (5.61) ng/mL, 47.25 (11.54) ng/mL and 55.35 (13.58) pg/mL, respectively. Demographic and surgical data of the patient groups were similar in terms of serum vitamin D concentrations (Table 1).

**Figure 3:**
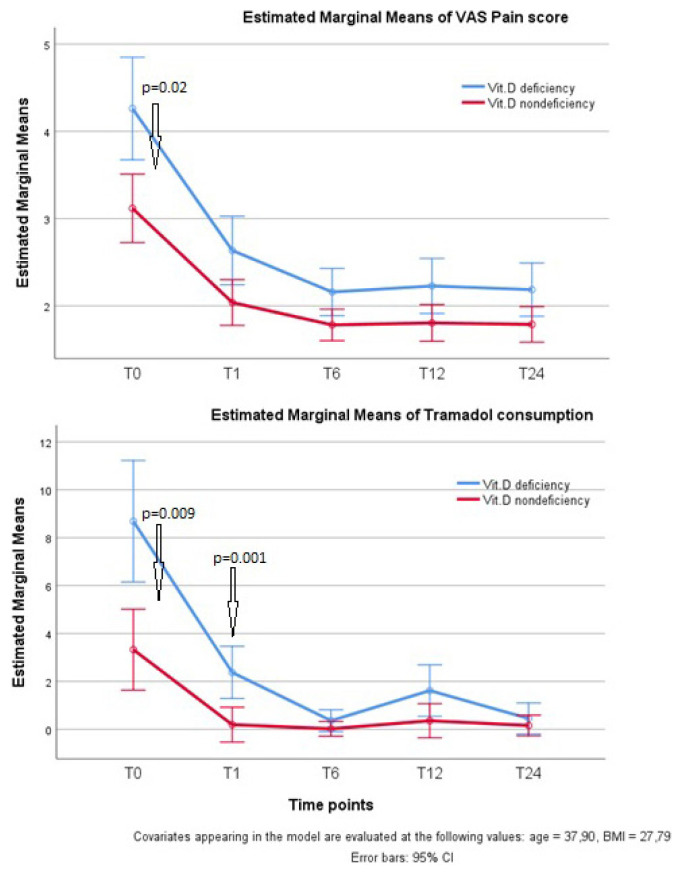
Time points of procedures.

Postoperative VAS pain scores were higher in the vitamin D deficiency group at all time points (T0–4), but differed significantly only at the T-0 time point (*p* = 0.020) (Figure 3). Tramadol consumptions were significantly higher in the vitamin D deficiency group only at T-0 and T-1 time points (*p* = 0.009, *p* = 0.001, respectively). Cumulative tramadol consumption was significantly higher in the vitamin D deficiency group (SD) (252.40 [13.93]) than in the nondeficiency group (243.82 [5.93]) (*p* = 0.005) (Table 1). Twenty-one (84.0%) patients in the vitamin D deficiency group and 42 (76.4) patients in the nondeficiency group required additional boluses of fentanyl intraoperatively (*p* = 0.439). In both the recovery room and the ward, none of the patients required rescue analgesia with morphine. Nausea was observed in 15% (12/80) patients, and vomiting in 6.3% (5/80). There were no significant differences between the groups with regard to postoperative side effects such as nausea and vomiting.

### 3.2. Analysis of clinical variables in terms of postoperative VAS (T-0) pain groups

When patients were divided into 2 groups according to VAS pain score at T-0 time point, serum vitamin D, DBP and VDR levels were significantly lower in patients with VAS ≥ 4 compared to patients with VAS < 4 (*p* = 0.009, *p* = 0.007, *p* = 0.024, respectively). In addition, the mean cumulative tramadol consumed in patients with VAS ≥ 4 (255.94 [9.79]) was significantly higher than in patients with VAS < 4 (240.21 [1.44]) (Table 2). The patients’ age, BMI, ASA physical status, and sex were similar between the groups.

### 3.3. Relationship between serum vitamin D concentrations, postoperative pain scores and tramadol consumption

There was a weak negative correlation between patients’ serum vitamin D levels and their postoperative pain scores (T0, T-1, T-2, T-4) as well as the amount of tramadol consumed (T-0 and T-1). However, there was a moderate negative correlation between serum vitamin D levels and cumulative tramadol consumption (*r* = −0.407) (Table 3). As expected, there was a very strong positive correlation between the serum vitamin D levels and the levels of DBP (*r* = 0.977) and VDR (*r* = 0.942) (*p* < 0.001).

### 3.4. Regression analysis of factors associated with postoperative pain score and cumulative tramadol consumption

According to multiple LR analysis, low serum vitamin D (*β* = −0.338) and high BMI (*β* = 0.263) were determined as independent risk factors for increased postoperative T-0 VAS pain score. These two factors accounted for 16.2% of the pain (Adj.R2 = 0.162). No correlation was found between the pain scores and age and sex (Table 4).

In the regression analysis, 69.5% (Adj.R2 = 0.695) of cumulative tramadol consumption was related to the severity of postoperative pain (*β* = 0.764) and vitamin D deficiency (*β* = −0.188). It was determined that 1 unit increase in VAS pain score caused a 4.8 mg increase in cumulative tramadol consumption, and 1 ng/mL increase in vitamin D level caused a 0.334 mg decrease in cumulative tramadol consumption. Age, sex, and BMI did not cause a significant change in the cumulative tramadol consumption (Table 4). When the same analysis was performed without including the VAS pain score in the regression model, 15.7% (Adj.R2 = 0.157) of cumulative tramadol consumption was found to be due to vitamin D deficiency.

## 4. Discussion

Although many clinical and experimental studies have shown the relationship between vitamin D levels and various pain states, its relationship with postoperative pain after cholecystectomy surgery has not been studied. The purpose of this prospective, observational study was to test the hypothesis that serum vitamin D concentrations affect the postoperative analgesic needs of patients undergoing cholecystectomy surgery. There were several important findings in this study. First, serum vitamin D levels were lower in patients with high postoperative pain scores. Second, postoperative pain scores and opioid analgesic consumption were significantly higher in patients with vitamin D deficiency compared to those with normal values. Third, there was a negative correlation between vitamin D levels, postoperative pain scores and opioid analgesic consumption. Finally, low serum vitamin D level was an independent risk factor for cumulative opioid analgesic consumption in the postoperative first 24 h.

Vitamin D is an important compound that is both absorbed and produced by the human body. For many years, it has been associated with many critical processes. Vitamin D insufficiency is continuously being investigated for the etiology of various diseases and for adverse postoperative outcomes [3,13,16]. Vitamin D deficiency is common in Turkey and its prevalence varies between 60% and 75% [17]. The present study was conducted for one year and the patients were under the age of 45. In our study, 25 (31.3%) patients had vitamin D deficiency, 70 (87.5%) had vitamin D insufficiency, and the mean serum vitamin D (SD) of the patients was 23.91 (5.61). Considering that vitamin D levels are lower in patients with biliary diseases and a history of cholecystectomy, our results are consistent with the literature [4–8].

Many previous studies have examined the relationship between vitamin D deficiency and various pain syndromes [9,18,19]. These studies have shown that low vitamin D levels are a risk factor for perceived pain [9,12.] A metaanalysis of 80 observational studies found that low vitamin D levels were associated with various types of pain [20]. However, the relationship between vitamin D levels and postoperative pain has been investigated in very few studies. In one of these studies, preoperative vitamin D deficiency was a risk factor for moderate to severe pain after knee arthroplasty [11], in another, perioperative serum vitamin D levels were reported to be a predictor for postoperative opioid consumption after major surgery [12]. These evidences in the literature reinforce our hypothesis that there is a relationship between postoperative pain and serum vitamin D levels.

Although the physiological mechanism of the relationship between vitamin D and pain has not yet been clearly explained, various hypotheses have been put forward. Studies have reported that the effects of vitamin D levels on pain perception are demonstrated via cortical, neuronal, immunological, hormonal, and anatomical changes [18–20]. Possible mechanisms of vitamin D in pain treatment include antiinflammatory and neuroimmunomodulatory effects, especially due to the decrease of PGE2 and T cell-mediated cytokine secretion [12,18,19] and modulating brain neurotransmitters (acetylcholine, dopamine and serotonin) similar to other steroids [18]. Moreover, both clinical and experimental studies have shown that vitamin D deficiency causes an increase in pain sensitivity as a result of hyperinnervation and hypersensitivity in nerve fibers [9,20]. It has also been reported that low vitamin D levels cause increased central neuronal sensitivity in chronic painful diseases such as fibromyalgia and migraine [9,21]. All these mechanisms show that vitamin D has an analgesic effect on various types of pain.

The results of studies investigating the relationship between vitamin D and pain are controversial. There are studies reporting that there is no relationship between vitamin D levels and postoperative pain scores and analgesic requirements [15,22,23]. In their retrospective study, Chester et al. [22] reported that preoperative and postoperative vitamin D levels were not associated with VAS pain scores in patients undergoing elective spinal surgery. Bose et al. [15] also did not find any relationship between preoperative vitamin D levels and pain scores/opioid consumption in the first postoperative 72 h in morbidly obese patients undergoing laparoscopic bariatric surgery. In addition, in the Cochrane review updated in 2015, it was reported that vitamin D supplementation used for chronic pain did not have a serious contribution to the treatment and randomized controlled studies were needed [24]. However, these studies have major limitations such as differences in reporting methodologies, retrospective design, and not standardizing postoperative pain management regimens. Unlike these studies, the strengths of our study are that it was a prospective observational study, all patients underwent a uniform surgical procedure, and that patients’ postoperative analgesia management regimens were standardized.

In our study, patients in both groups had adequate pain relief as evidenced by similar postoperative pain scores (except T-0). Accordingly, these similar results in pain scores were achieved at the expense of significantly higher opioid analgesic consumption in the vitamin D deficient group. Patients in both groups had immediate access to tramadol with IV PCA so that they could achieve adequate analgesic effect. These results suggest that high serum vitamin D levels may have a dose-sparing effect on postoperative opioid analgesic consumption. Studies in the literature have shown that vitamin D supplements can increase the efficacy of opioids by reducing opioid tolerance [12,23]. However, further prospective clinical studies investigating whether prophylactic vitamin D supplementation can reduce postoperative opioid need in surgical patients are needed.

One of the major findings of our study was that low serum vitamin D level was an independent risk factor for postoperative opioid analgesic consumption. In previous studies, low vitamin D levels have been associated with the amount and duration of short-term opioid use in palliative cancer patients [23,25]. A recent cohort study aimed to determine the relationship between serum vitamin D levels and long-term opioid use and opioid use disorder in patients undergoing major surgical procedures. In that study, the authors reported that vitamin D deficiency was a predictor for postoperative opioid consumption, and that those with vitamin D deficiency had a higher risk of opioid use disorder [12]. In our study, however, low vitamin D levels were not a predictor for postoperative opioid consumption and were only found as an independent risk factor. Our study can pave the way for subsequent randomized controlled clinical trials investigating the effects of vitamin D supplements on postoperative pain. In the future, detailed pathophysiological studies are needed to clarify the role of patient’s vitamin D status in postoperative pain.

Detailed examination of our results showed that despite exclusion of patients with BMI >35 kg/m2, it was found that high BMI was a risk factor for VAS-T0 pain score in the regression analysis, but not for cumulative tramadol consumption. However, BMI values did not differ significantly between vitamin D groups. There are conflicting results in the literature regarding the relationship between BMI and pain. In a metaanalysis of 33 studies examining negative predictors associated with postoperative pain control, it was reported that higher BMI was an independent risk factor for postoperative pain [26]. On the other hand, in another study, no relationship was found between BMI and acute postoperative pain scores [27]. According to our study, although BMI was a risk factor for postoperative high pain scores, the effect of BMI on pain scores still remains a controversial and researched issue. In the same metaanalysis, it was also reported that younger age, female sex, smoking, history of depressive and anxiety symptoms, sleep difficulties, presence of preoperative pain, and use of preoperative analgesia were independent risk factors for postoperative pain [26]. In our study, age and sex were not found to be risk factors for postoperative pain, and other risk factors were not examined. In another study, while age is associated with slightly lower postoperative pain scores, sex is not associated [27].

### 4.1. Limitations of the study

Our study has several limitations. First, no adjustments were made for seasonal variation and climate-related factors affecting serum vitamin D levels during the study period. However, blood samples were taken from all patients before induction of anesthesia to determine their current serum vitamin D levels. In future studies, the relationship between seasonal vitamin D levels and postoperative pain can be investigated. Our second limitation is that various factors that have been shown to affect acute postoperative pain (anxiety, depression, smoking, socioeconomic status, education) were not investigated. Future research may focus on analyzing effects of various pain predictors in conjunction with vitamin D deficiency on postoperative pain with a larger patient population. Finally, our study was conducted in a single institution and only included patients undergoing laparoscopic cholecystectomy. Thus, it cannot be generalized to all patients undergoing surgery. Nevertheless, in the light of the literature, we think that the present study addresses an important issue in the field of pain.

## 5. Conclusion

In conclusion, our study showed that preoperative low serum vitamin D levels were associated with an increase in acute postoperative pain scores and opioid analgesic consumption in patients undergoing laparoscopic cholecystectomy. These findings may be useful for managing postoperative pain in patients with vitamin D deficiency. We believe that it will be useful to apply preventive treatments for postoperative pain in patients with vitamin D deficiency. Larger clinical studies are needed to confirm the relationship between serum vitamin D levels and postoperative pain.

## Figures and Tables

**Table 1:** Demographic and clinical characteristics of the groups according to serum vitamin D concentrations.

Variables	Vitamin D deficiency group* (n = 25)		Vitamin D nondeficiency group (n = 55)		Total (n = 80)		
	Mean (SD)		Mean (SD)		Mean (SD)		*p-*value
Age (year)	39.20	(3.46)	37.31	(4.13)	37.90	(4.01)	0.050^b^
BMI (kg/m)	27.42	(4.34)	28.60	(3.63)	27.79	(4.15)	0.240^b^
Surgical time (min)	71.00	(13.39)	72.18	(10.44)	71.81	(11.37)	0.569^b^
Sex, male/female, (%)	6/19 (24.0/76.0)		20/35 (36.4/63.6)		26/54 (32.5/67.5)		0.274^c^
ASA physical status, I/II/III (%)	8/15/2 (32.0/60.0/8.0)		23/28/4 (41.8/50.9/7.3)		31/43/6 (38.8/53.8/7.5)		0.703^c^
VAS < 4/VAS ≥ 4 patients (%)	10/15 (40.0/60.0)		38/17 (69.1/30.9)		48/32 (60.0/40.0)		0.014^c^
Patients requiring fentanyl bolus intraoperatively, (%)	21 (84.0)		42 (76.4)		63 (78.8)		0.439^c^
**VAS pain scores**							
Pain VAS score-T0	4.08	(1.63)	3.20	(1.48)	3.47	(1.57)	0.020^b^
Pain VAS score-T1	2.56	(1.19)	2.07	(0.88)	2.23	(1.01)	0.179^a^
Pain VAS score-T6	2.12	(0.83)	1.80	(0.59)	1.90	(0.69)	0.053
Pain VAS score-T12	2.16	(0.99)	1.84	(0.69)	1.94	(0.80)	0.094
Pain VAS score-T24	2.08	(1.00)	1.84	(0.71)	1.91	(0.81)	0.217
**Tramadol consumption**							
Tramadol consumption-T0 (mg)	8.00	(7.64)	3.64	(5.89)	5.00	(6.75)	0.009^a^
Tramadol consumption-T1 (mg)	2.40	(4.36)	0.18	(1.35)	0.88	(2.84)	0.001^a^
Tramadol consumption-T2 (mg)	0.40	(2.00)	0.00	(0.00)	0.12	(1.12)	0.138^a^
Tramadol consumption-T3 (mg)	1.60	(3.74)	0.36	(1.89)	0.75	(2.65)	0.053^a^
Tramadol consumption-T4 (mg)	0.40	(2.00)	0.18	(1.35)	0.25	(1.57)	0.568
24h cumulative tramadol consumption (mg)	252.40	(13.93)	243.82	(5.93)	246.50	(9.95)	0.005^a^
Vitamin D (ng/mL)	16.77	(1.74)	27.15	(3.23)	23.91	(5.61)	<0.001^a^
VDBP (ng/mL)	33.91	(3.32)	53.31	(8.37)	47.25	(11.54)	<0.001^a^
VDR (pg/mL)	40.23	(4.74)	62.26	(10.26)	55.38	(13.58)	<0.001^a^

ASA: American Society of Anesthesiologists; SD: standard deviation; BMI: body mass index; VDBP: vitamin D binding protein; VDR: vitamin D receptor; VAS: visual analog scale.

* Defined as serum 25-hydroxyvitamin D concentration < 20 ng/mL.

a Mann-Whitney U test,

b independent sample t-test,

c chi-square test.

**Table 2:** Clinical variables according to postoperative VAS pain groups.

	VAS < 4 patients (n = 48)		VAS ≥ 4 patients (n = 32)		
	Mean	(SD)	Mean	(SD)	*p-*value
Age (year)	38.29	(4.01)	37.31	(4.00)	0.287^b^
BMI (kg m^−2^)	27.06	(3.78)	28.27	(4.35)	0.204^b^
ASA physical status, I/II/III (%)	16/28/4 (33.3/58.3/8.3)		15/15/2 (46.9/46.9/6.3)		0.475^c^
Sex, male/female (%)	19/29 (39.6/60.4)		7/25 (21.9/78.1)		0.098^c^
24h Cumulative tramadol consumption (mg)	240.21	(1.44)	255.94	(9.79)	< 0.001^a^
Vitamin D (ng mL^−1^)	25.30	(4.88)	21.81	(6.05)	0.009^a^
VDBP (ng mL^−1^)	50.05	(10.48)	43.05	(11.94)	0.007^b^
VDR (pg mL^−1^)	58.15	(12.52)	51.22	(14.23)	0.024 b

ASA: American Society of Anesthesiologists; SD: standard deviation; BMI: body mass index; VDBP: vitamin D binding protein; VDR: vitamin D receptor; VAS: visual analog scale.

a Mann-Whitney U test,

b independent sample t-test,

c chi-square test.

**Table 3:** Correlation analysis between serum vitamin D level, postoperative pain scores and tramadol consumption.

	Vitamin D	VAS-T0^a^	VAS-T1^a^	VAS-T2^a^	VAS-T3^a^	VAS-T4^a^	Tramadol consumption-T0	Tramadol consumption-T1	Tramadol consumption-T2	Tramadol consumption-T3	Tramadol consumption-T4
VAS-T0^a^	−0.274*	1.000									
VAS-T1^a^	−0.253*	0.732**	1.000								
VAS-T2^a^	−0.222*	0.580**	0.615**	1.000							
VAS-T3^a^	−0.163	0.522**	0.519**	0.796**	1.000						
VAS-T4^a^	−0.220*	0.462**	0.421**	0.637**	0.745**	1.000					
Tramadol consumption-T0	−0.323**	0.883**	0.632**	0.444**	0.440**	0.404**	1.000				
Tramadol consumption-T1	−0.319**	0.454**	0.520**	0.484**	0.334**	0.354**	0.495**	1.000			
Tramadol consumption-T2	−0.206	0.131	0.184	0.224*	0.205	0.165	0.084	0.363**	1.000		
Tramadol consumption-T3	−0.189	0.172	0.235*	0.366**	0.518**	0.418**	0.141	0.248*	0.395**	1.000	
Tramadol consumption-T4	−0.059	0.072	0.122	0.159	0.248*	0.296**	0.001	0.234*	−0.018	−0.046	1.000
24h Cumulative tramadol consumption	−0.407**	0.873**	0.647**	0.516**	0.509**	0.463**	0.886**	0.738**	0.381**	0.389**	0.138

* Correlation is significant at the 0.05 level (2-tailed).

** Correlation is significant at the 0.01 level (2-tailed). VAS: visual analog scale.

a VAS scores, vitamin D and tramadol consumption by Spearman’s rho correlation,

Note: Since there is a very strong positive correlation between vitamin D and vitamin D binding protein *(r* = 0.977) and vitamin D receptor (*r* = 0.942), these two parameters were not included in the correlation table (*p* < 0.001).

**Table 4:** Analysis of factors associated with postoperative pain score and cumulative tramadol consumption.

Dependent variable (n = 80)	Unstandardized coefficients	95.0% confidence interval for B		Standardized coefficients	t	*p-*value
	B	Lower bound	Upper bound	β		
**VAS-T0**^a^ Adj.R^2^=0.162						
(Constant)	11.190	7.068	15.312		5.407	< 0.001
Vitamin D	−0.095	−0.154	−0.036	−0.338	−3.210	0.002
BMI	0.100	0.179	0.021	0.263	2.529	0.014
**24h cumulative tramadol consumption**^b^ Adj.R^2^ = 0.695						
(Constant)	237.705	230.745	244.665		68.010	< 0.001
Vitamin D	−0.334	−0.563	−0.105	−0.188	−2.903	0.005
VAS-T0	4.829	4.013	5.645	0.764	11.779	< 0.001

a Independent variables; age, sex (dummy), BMI, surgical time, Vitamin D

b Independent variables; age, sex (dummy), BMI, surgical time, Vitamin D, VAS-T0.

BMI: body mass index; VAS: visual analog scale.
